# Reactive Arthritis Post-SARS-CoV-2

**DOI:** 10.7759/cureus.18139

**Published:** 2021-09-20

**Authors:** Faizal Ouedraogo, Rachita Navara, Rusha Thapa, Kunj G Patel

**Affiliations:** 1 Department of Medicine, University of Maryland Capital Region Health, Largo, USA; 2 Division of Cardiology, Washington University School of Medicine, St. Louis, USA; 3 Medicine, Nepal Medical School, Kathmandu City, NPL; 4 Department of Physical Medicine and Rehabilitation, Barnes-Jewish West County Hospital, St. Louis, USA

**Keywords:** reactive arthritis, polyarthritis, covid-19, sars-cov-2, case report

## Abstract

Reactive arthritis (ReA) following bacterial infection from the urogenital and gastrointestinal tract is widely described but is not typical post-viral infections. This report presents the second case of ReA after severe acute respiratory syndrome coronavirus 2 (SARS-CoV-2) infection in the United States. A 45-year-old black male with chronic low back pain was hospitalized for 45 days with coronavirus disease 2019 (COVID-19), complicated due to the development of multiorgan failure managed with intubation, extracorporeal membrane oxygenation, and hemodialysis. He was subsequently discharged to an acute rehabilitation facility where he complained of new-onset pain in his shoulders, left elbow, and left knee three weeks after a negative SARS-CoV-2 test. He was readmitted from his acute rehabilitation facility due to recurrent fever and the development of a swollen, warm left knee. Laboratory studies at readmission showed elevated inflammatory markers, negative extensive infectious disease workup, and aseptic inflammatory left knee synovial fluid without crystals. Testing returned negative for most common antibodies seen in immune-mediated arthritides (e.g., rheumatoid arthritis, systemic lupus erythematosus), as well as for common respiratory and gastrointestinal tract pathogens responsible for viral arthritis. The multidisciplinary inpatient medical team deemed the clinical presentation and laboratory findings most consistent with ReA. The patient received a course of oral corticosteroids, followed by a second course due to the recurrence of symptoms weeks after initial treatment and recovery. The current body of medical literature on SARS-CoV-2 pathophysiology supports plausible mechanisms on how this infection may induce ReA. Such a scenario should be considered in the differential of COVID-19-recovered patients presenting with polyarthritis as prompt steroid treatment may help patient recovery.

## Introduction

Patients with reactive arthritis (ReA) typically present with aseptic oligoarthritis one to six weeks after extra-articular infection, usually of gastrointestinal or urogenital origin [1]. Although less commonly reported in the medical literature, the respiratory tract is also a site of underlying infection that can lead to ReA. Only a few cases of ReA in the context of viral infections have been reported [2]. Since the beginning of the severe acute respiratory syndrome coronavirus 2 (SARS-CoV-2) pandemic, epidemiologic data have allowed scientists to build accurate clinical case definitions of coronavirus disease 2019 (COVID-19). However, some questions remain partially unanswered when it comes to the post-infection evolution. We present a case of ReA following SARS-CoV-2 infection, adding to the building literature detailing the post-COVID-19 manifestations.

## Case presentation

A 45-year-old black male with a family history of hypertension and prostate cancer, a personal history of chronic low back pain status post spinal fusion, and a recent 45-day hospitalization for COVID-19 pneumonia developed a new-onset polyarthritis three days after his discharge to acute inpatient rehabilitation. His COVID-19 syndrome consisted of a five-day history of productive cough and fever with a real-time polymerase chain reaction (RT-PCR) of his nasal swab positive for SARS-CoV-2. After admission, his COVID-19 pneumonia was managed with azithromycin, ceftriaxone, hydroxychloroquine, and tocilizumab. He later developed multiorgan failure during his hospitalization, which required 21 days of intubation with prone positioning, 16 days of veno-venous extracorporeal membrane oxygenation, and continuous renal replacement therapy. He had two negative repeat testing for SARS-CoV-2 on days 27 and 31.

**Figure 1:**
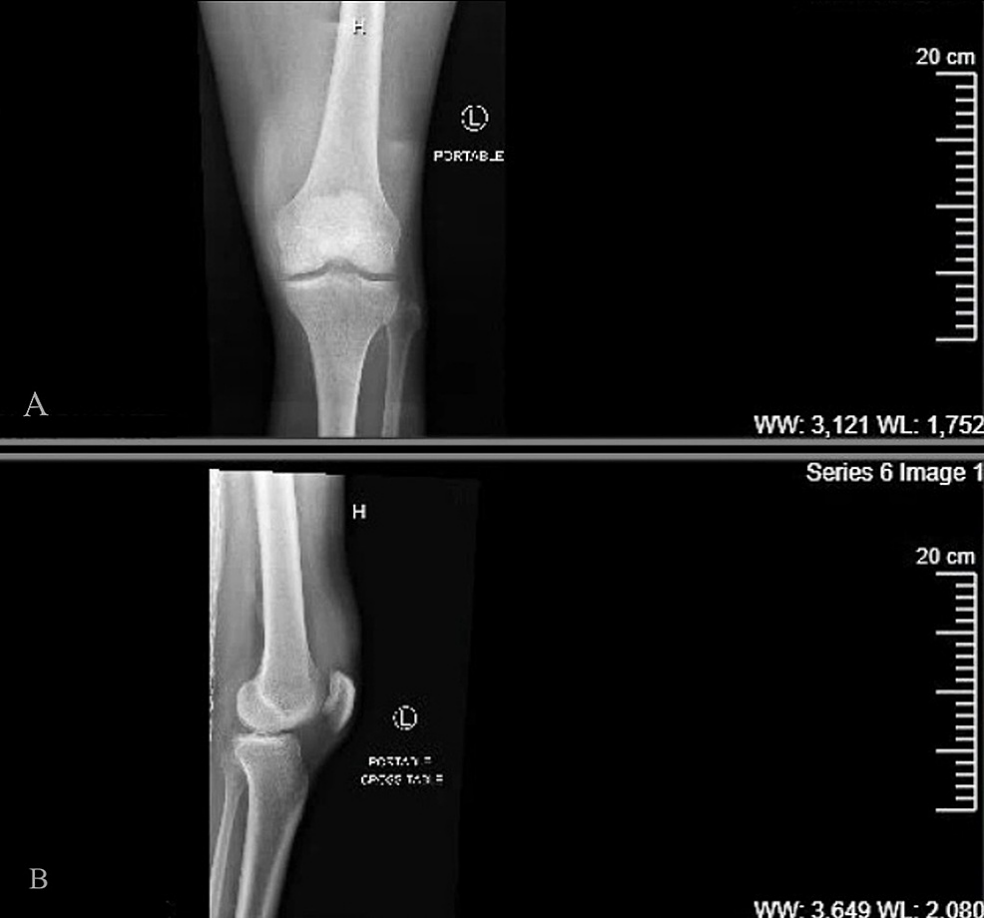
X-ray of the left knee. (A) Anteroposterior view. (B) Lateral view.

On day 48, while discharged to our rehabilitation hospital, he complained of significant pain in his shoulders, left elbow, and left knee. He was readmitted on day 52, given worsening limiting pain and recurrence of fever. At readmission, he was febrile to 101.1°F, tachycardic to 119 beats per minute, and tachypneic to 23 beats per minute. He was noted to have an erythematous, warm, swollen left knee, as shown in the X-ray (Figure 1). He did not have rash, conjunctivitis, or urethritis.

Pertinent readmission labs were notable for elevated lactate at 2.3 mmol/L, erythrocyte sedimentation rate at 136 mm/hour, C-reactive protein at 178 mg/dL, and white blood cell (WBC) count at 17.5 × 10^3^/uL. Repeat cultures (blood and urine), transthoracic echocardiography, and lower extremity duplex were all negative. Left knee arthrocentesis yielded 11 × 10^3^/uL WBC with 94% polymorphonuclears, no microscopic evidence of monosodium urate (MSU) and calcium pyrophosphate, and no microorganisms in the synovial fluid culture. Antigenic tests for gonorrhea, chlamydia, hepatitis B, *Clostridioides difficile*, and HIV were negative. Serologic tests for rheumatoid factor, anti-cyclic citrullinated peptide antibodies, cytomegalovirus, Epstein-Barr virus, enterovirus, parvovirus B19, and *Treponema pallidum* were negative. The multidisciplinary medical team diagnosed the patient with ReA. Given his recent renal impairment, they started him on a tapered dose of oral corticosteroid with significant improvement in pain and resolution of fever.

## Discussion

Both the definition and the diagnosis of ReA have suffered from a lack of consensus among experts in the medical community. Some authors reserve the term ReA for post-inflammatory arthritis caused by common bacteria, while other forms of arthritis caused by other pathogens are referred to as post-infectious arthritis [3]. In 1969, ReA was defined as aseptic arthritis following remote infection, usually from the urogenital or gastrointestinal tract [4]. Per this more liberal definition, experts at the 1999 international workshop on ReA in Germany offered a list of causative pathogens and tentative classification criteria [5]. Although there are no clearly established criteria for the diagnosis or classification of ReA, the diagnosis is usually made using clinical and microbiological findings [6].

Hypothetically, any microorganism can trigger the pathophysiology of ReA. In addition to the most commonly encountered bacteria, there have been reports of fungal [7], parasitic [8], and viral [2] pathogens in ReA. Since the beginning of the COVID-19 pandemic, few cases of SARS-CoV-2 ReA have been reported. While caring for the patient presented, three additional cases were reported worldwide, including one in the United States. The case we present here, the second in the United States, has unique features regarding the preceding infection course, the outcomes, and therapeutic choices (Table 1) [9-11]. Indeed, our patient had a more severe COVID-19 syndrome with more extended hospitalization and rapid deterioration with early implementation of prolonged advanced supportive care. This might have played a role in the outcome of his ReA, which was marked by a remission recurrence pattern despite using systemic steroids with a looming diagnosis of chronic ReA.

**Table 1:** Reported cases of ReA following SARS-CoV-2 infection. MTP: metatarsophalangeal; NSAIDs: non-steroidal anti-inflammatory drugs; PT/OT: physical therapy/occupational therapy; ECMO: extracorporeal membrane oxygenation; SARS-CoV-2: severe acute respiratory syndrome coronavirus 2; ReA: reactive arthritis

Cases	Country Age/Gender	COVID-19 hospital stay	Advanced supportive care	ReA clinical presentation	ReA treatment	Clinical course
Ono et al. [9]	Japan 50/Male	20 days	7-day intubation	Oligoarthritis (bilateral ankles) and enthesitis (right Achilles tendon)	NSAIDs, intraarticular corticosteroids	Moderate improvement
Saricaoglu et al. [10]	Turkey 73/Male	22 days	No	Polyarthritis: Left MTP of the hallux, proximal, and distal interphalangeal of the second toe on the right foot	NSAIDs	Resolved
Danssaert et al. [11]	United States 37/Female	0 day	No	Monoarthritis (hand), tendonitis (flexor and extensor hand)	Diclofenac gel, gabapentin, and hydromorphone	Enthesitis persisted for the next 4 weeks managed with splinting, OT, and tramadol
This case	United States 45/Male	45 days	21-day intubation, 16-day ECMO, and dialysis	Polyarthritis (knee, elbows, shoulder)	Oral prednisone	Recurrence of polyarthritis managed with second dose taper of steroid + PT/OT

Our patient presented with acute polyarthritis three weeks after a negative RT-PCR test for SARS-CoV-2. His symptomatology was deemed less likely originating from other potential causes in the differential after thorough diagnostic and etiologic workup. Although there was bacteremia during hospitalization, a negative extensive infectious disease workup ruled out septic arthritis. Furthermore, Gram staining, PCR, and culture of synovial fluid did not yield any microorganisms. Titration of the most common antibodies in immune-mediated arthritides and PCR for common respiratory and gastrointestinal tract pathogens responsible for viral arthritis yielded unremarkable results. Although our patient had an isolated episode of crystalline negative podagra 12 years ago, the current knee arthrocentesis did not reveal MSU crystals, and his plasma uric acid was normal. Despite chondrocalcinosis on knee radiography, there was no evidence of calcium pyrophosphate crystals in synovial fluid. We ruled out viral arthritis from other common viral pathogens, but we still cannot omit the possibility of SARS-CoV-2-induced arthritis. However, given the timeline of occurrence, it seems less likely. Indeed, cases of delayed onset and prolonged arthritis in viral infections have been frequently reported with alphaviruses and hepatitis C virus, none have been reported for coronaviruses [12]. Studies show that typical musculoskeletal symptoms associated with SARS-CoV-2 tend to present with acute myalgia and arthralgia rather than delayed clinical arthritis [13].

The interplay between host antimicrobial factors with the theory of unbalanced Th1/Th2 cytokine secretion and genetic factors with the human leukocyte antigen (HLA) B27 theory in the pathophysiology of ReA [14] may explain how the SARS-CoV-2 virus elicits ReA. Indeed, microorganisms in ReA are thought to trigger an inflammatory response from the host through antigenic cross-reactivity [15]. Molecular mimicry is the mechanism through which it occurs and has been well documented for bacterial pathogens commonly seen in ReA [15]. Although not clearly understood, studies have shown that the pathogenesis of viral-induced arthritis involves some degree of molecular mimicry, bystander activation, and viral persistence, depending on the virus [16]. Moreover, viral-induced immunoreactivity plays a role in the pathogenesis of rheumatic and autoimmune diseases parallel to other host-specific factors such as genetic susceptibility [17]. Therefore, the SARS-CoV-2 virus can elicit ReA.

Another potential mechanism of SARS-CoV-2-induced ReA includes the interaction of the virus with its angiotensin-converting enzyme 2 (ACE2) receptors in the gastrointestinal tract. Studies have shown that such an interaction downregulates ACE2 expression, limiting its functions [18] in gut immune and microbial homeostasis [19]. In genetically susceptible individuals, it is suggested that such dysregulation of the gut microbiome plays a role in the pathogenesis of spondyloarthropathies such as ReA [20]. Besides the misfolding and the arthritogenic postulates [14], dysbiosis [20] is another hypothesis on the role of HLA B27 in ReA. Underlying the latter theory is the body of research on the regulatory functions of enterocytes ACE2 receptors. Despite the plausibility of this theory, more studies are needed to understand how this mechanism may elicit ReA and the timeframe required to do so.

The body of literature on the manifestations following COVID-19 is expanding and has ramifications in many disciplines. The medical community is still grasping the full picture of the aftermath of SARS-CoV-2 infection among recovered patients. Awareness about the possibilities of ReA in the immediate course of recovery from COVID-19 is essential as it may impede physical and occupational therapies (PT/OT), which are crucial in the recovery process. Moreover, in patients who have had a severe COVID-19 course and required intensive care management, ReA may coexist with the active or lingering symptoms of critical illness myopathy to further limit optimal gain from PT/OT. It points to the importance of an adequate multidisciplinary team involving rheumatologists, physiatrists, pain physicians, and PT/OT for efficient management in such scenarios. Although seldom involved in the management, the expertise of physiatrists/pain specialists can make a significant contribution. For instance, when anti-inflammatories are contraindicated, or there is persisting recurrent pain despite standard of care, the use of multimodal pain management is an available alternative. Among the cases reported, including this one, individuals benefited from physiatrist care in only two instances.

## Conclusions

This case raises awareness of the potential post-inflammatory manifestations that can plague the recovery following COVID-19 infection. It adds to the growing literature emphasizing the need for clinicians to consider ReA in arthritic presentations after COVID-19. On the other hand, it sheds light on the need for more research to assess the alleged role of viral etiologies as potential pathogens in the occurrence of ReA.
